# Is Craniosacral Therapy Effective? A Systematic Review and Meta-Analysis

**DOI:** 10.3390/healthcare12060679

**Published:** 2024-03-18

**Authors:** Luis Ceballos-Laita, Edzard Ernst, Andoni Carrasco-Uribarren, Sara Cabanillas-Barea, Jaime Esteban-Pérez, Sandra Jiménez-del-Barrio

**Affiliations:** 1 Department of Surgery, Ophthalmology and Physiotherapy, University of Valladolid, 42004 Soria, Spain; luis.ceballos@uva.es (L.C.-L.); jaime.esteban.perez@uva.es (J.E.-P.); 2 Complementary Medicine, University of Exeter, Exeter EX4 4SB, UK; e.ernst@exeter.ac.uk; 3 Faculty of Medicine and Health Sciences, International of Cataluña University, 8195 Sant Cugat del Vallés, Spain; acarrasco@uic.es (A.C.-U.); scabanillas@uic.es (S.C.-B.)

**Keywords:** complementary therapies, osteopathy, systematic review, meta-analysis

## Abstract

Objectives: The aim of this study was to evaluate the clinical effectiveness of craniosacral therapy (CST) in the management of any conditions. Methods: Two independent reviewers searched the PubMed, Physiotherapy Evidence Database, Cochrane Library, Web of Science, and Osteopathic Medicine Digital Library databases in August 2023, and extracted data from randomized controlled trials (RCT) evaluating the clinical effectiveness of CST. The PEDro scale and Cochrane Risk of Bias 2 tool were used to assess the potential risk of bias in the included studies. The certainty of the evidence of each outcome variable was determined using GRADEpro. Quantitative synthesis was carried out with RevMan 5.4 software using random effect models. Data Synthesis: Fifteen RCTs were included in the qualitative and seven in the quantitative synthesis. For musculoskeletal disorders, the qualitative and quantitative synthesis suggested that CST produces no statistically significant or clinically relevant changes in pain and/or disability/impact in patients with headache disorders, neck pain, low back pain, pelvic girdle pain, or fibromyalgia. For non-musculoskeletal disorders, the qualitative and quantitative synthesis showed that CST was not effective for managing infant colic, preterm infants, cerebral palsy, or visual function deficits. Conclusions: The qualitative and quantitative synthesis of the evidence suggest that CST produces no benefits in any of the musculoskeletal or non-musculoskeletal conditions assessed. Two RCTs suggested statistically significant benefits of CST in children. However, both studies are seriously flawed, and their findings are thus likely to be false positive.

## 1. Introduction

Craniosacral therapy (CST) is defined as an intervention based on a gentle touch that allegedly releases restrictions in any tissues influencing the craniosacral system [1]. It has been considered as complementary and alternative medicine by the World Health Organization (WHO) and has been included in the Benchmarks for Osteopathic Education of the WHO [2].

Osteopathy is frequently used by patients with conditions such as back pain, neck pain, fibromyalgia, digestive disorders, or infantile colic [3,4,5]. International surveys have reported that 23% to 90% of osteopaths use CST. Specifically in Europe, between 70% and 89% of the interviewed osteopaths use CST always or often [4,6,7,8,9]; and 23% to 46% use it as a first-line treatment [10,11]. The relationship between the craniosacral system and the mentioned diseases has been theoretically based on implausible and unproven anatomical claims and connections [12], which means that no real relationship has been established, making the use of CST less than plausible. 

The biological model of CST is commonly known as the “primary respiratory mechanism” (PRM) or “craniosacral mechanism”. It assumes that the cranial structures present intrinsic mobility and can be detected by manual palpation [13]. These anatomical connections include minuscule or even nanoscopic motions of the osseous and membranous movements of the skull and its contents [14]. The underlying assumption is that movement in the cranial structures causes rhythmic movements of the cerebrospinal fluid from the cranium to the sacrum and specific changes in the dural membranes, as well as cranial and sacral bones [15]. To date, no evidence is available to suggest any mobility of the cranial bones. Under normal conditions, the cranial sutures fuse completely between the ages of 13 and 18 years, which means that adult cranial bones are fused [16,17]. In addition, the reliability of the palpation of the PRM is not supported by sound evidence. Guillaud et al. [18] reviewed nine studies testing the intra- and inter-rater reliability. All had a high risk of bias and failed to demonstrate that the palpation of the PRM is a valid diagnostic method. 

Hestbaeck et al. [5] pointed out that despite the lack of benefits found in favor of CST in previous clinical trials and the low methodological quality presented in some of them, the use of osteopathy is supported by the interest of the patients in such therapies. However, the popularity of a therapy is a poor indicator of its effectiveness, and all interventions must demonstrate their true value through well-designed clinical trials. 

The clinical effectiveness of CST has been tested in numerous clinical trials and summarized in several previous systematic reviews and meta-analyses. Three systematic reviews concluded that there was insufficient evidence to support the application of CST in patients with headache disorders, low back pain, lateral epicondylitis, fibromyalgia, visual alterations, asthma, attention deficit hyperactive disorders, infantile colic, preterm infants, and cerebral palsy mainly because the studies included were seriously flawed [1,15,18]. However, these systematic reviews also included studies not related to the clinical effectiveness of CST. Only Haller et al. [19] conducted a systematic review and meta-analysis suggesting that CST was effective in managing chronic pain in different musculoskeletal conditions. However, the combination of different conditions, such as fibromyalgia and neck pain, in the same forest plot decreases the validity of these results for combining populations that are not homogeneous. 

Since the publication of these papers, several new randomized clinical trials (RCTs) of CST have emerged. The aim of this systematic review and meta-analysis is to evaluate the totality of the evidence for or against the clinical effectiveness of CST in the management of any conditions. 

## 2. Materials and Methods

### 2.1. Study Design

A systematic review with meta-analysis was designed following the PRISMA statement and the Cochrane recommendations [20]. The protocol of this review was prospectively registered in PROSPERO (identification number CRD42023454524).

### 2.2. Search Strategy

The bibliographical searches were carried out in PubMed, the Physiotherapy Evidence Database (PEDro), Cochrane Library, Web of Science (WOS), and Osteopathic Medicine Digital Library (OSTMED) from inception to August 2023. Medical Subject Heading (MeSH) terms and grey terms were applied in the search strategy: osteopathic manipulation, osteopathic cranial manipulative medicine, cranial osteopathic manipulative medicine, cranial osteopathy, and craniosacral therapy, among others. The search strategy used in each database is shown in Appendix A. The reference lists of the included studies and the above-mentioned previous systematic reviews were hand-searched. 

### 2.3. Eligibility Criteria and Study Selection

Studies were considered if they: included patients with musculoskeletal or non-musculoskeletal conditions; applied CST in isolation or in addition to standard care; compared the craniosacral intervention to a sham control or standard care intervention; reported variables related to the clinical effectiveness; and were designed as randomized controlled trials. Studies were excluded if they: included healthy participants; applied a multimodal intervention or comparator in which the effects of CST could not be extrapolated; reported no clinical outcomes (but only variables such as heart rate, skin conductance, or breathing rate), or the outcome measures were not quantified using validated instruments. 

The reference lists retrieved from each database were exported to Mendeley to remove duplicates. Two authors (LC and AC) independently reviewed the title and abstract of each retrieved study to determine its potential eligibility. The studies that met the eligibility criteria were assessed in full text by the same authors. A third author (SJ) was consulted in case of discrepancies. 

### 2.4. Data Extraction

The data extraction was performed independently by the two authors using a predetermined sheet adapted from the Cochrane Collaboration. The data extracted were the characteristics of the population (sex ratio, mean age, and diagnosis), type of interventions (session duration, sessions per week, and total number of sessions), outcome variables, and results. Data were analyzed using a qualitative and quantitative synthesis. 

### 2.5. Methodological Quality, Risk of Bias, and Certainty of Evidence

The methodological quality, risk of bias, and certainty of evidence were assessed using the PEDro scale, Cochrane Risk of Bias 2 tool, and GRADEPro, respectively. The same authors independently performed the assessments.

The PEDro scale is an 11-item scale based on a Delphi list to assess the methodological quality of clinical trials [21]. A score of 7 or above was considered “high” quality, 5 to 6 was considered “fair” quality, and 4 or below was considered “poor quality”. The first item of the PEDro scale (eligibility criteria) is related to external validity and was not considered in the total score.

The Risk of Bias 2 tool was used to determine the potential risk of bias in the RCTs and classified them as low, unclear, or high risk, based on five domains. The combination of the previously mentioned five items was used to determine the overall risk of bias rating for the entire study [22]. 

GRADEPro categorizes the certainty of evidence as “high”, “moderate”, “low”, or “very low”. A moderate or high certainty indicates that we are moderately or very confident in the effect estimate. A low certainty means that the true effect can be substantially different from the estimated one, and very low certainty means that the true effect is likely to be substantially different from the estimated effect. 

The certainty of evidence for the meta-analysis was downgraded based on the presence of certain factors, including the risk of bias, inconsistency of the results, indirectness of evidence, and imprecision. The risk of bias was downgraded by one level or two levels when 25% or 50% of the subjects included in a study originated from clinical trials with a high risk of bias: lack of random allocation and/or sample size calculation of participants, allocation concealment, and/or personnel blinding of outcome assessors. Inconsistency of results was downgraded by one or two levels when the I^2^ was ≥50 or ≥75 [23]. Indirectness of evidence was downgraded by one level if different populations, interventions, or comparators were included, and imprecision was downgraded by one or two levels if the number of participants in the comparison was less than 100 or ≤ 30 individuals [24]. 

### 2.6. Data Synthesis and Analysis

A qualitative synthesis of the results was conducted and, whenever this was possible, a quantitative synthesis (meta-analysis) was carried out using the RevMan 5.4 software. 

Meta-analyses were performed if at least two studies were sufficiently homogeneous. Studies were considered homogeneous if they applied a common intervention, measured a common outcome, and included the same population. When a three-arm study was included, the data from the repeated groups were divided to avoid duplicate data [25]. Outcomes were analyzed based on the post-intervention means and standard deviations (SDs) by calculating the mean difference (MD) when RCTs used the same scale, or standardized mean difference (SMD) when they used different scales, with 95% coefficient intervals (CIs). SMD classifies the effects estimates as small (SMD at least 0.2 but less than 0.5), medium (SMD from 0.5 to less than 0.8), or large (SMD 0.8 or greater) [26]. Significance was set at a *p*-value < 0.05.

A random-effect meta-analysis was performed when combinations of intervention effects were based on the assumption that the studies are not all estimating the same intervention effect [27].

To detect publication bias, Begg and Egger tests were conducted using EPIDAT 3.1. Funnel plots were not reported because fewer than 10 trials were available. 

## 3. Results

**Figure 1:**
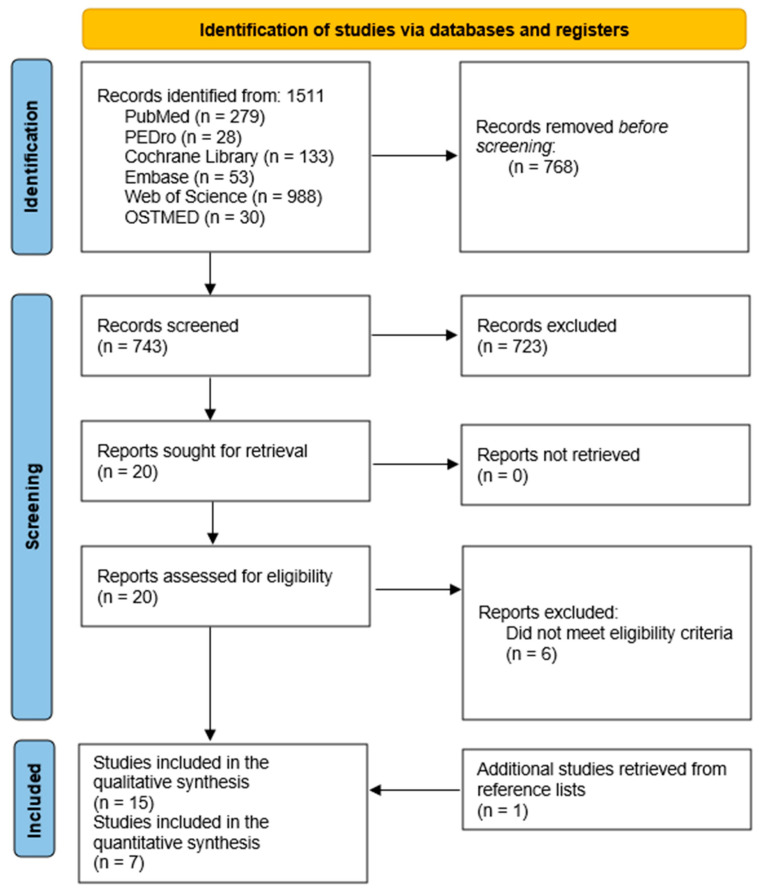
Flowchart diagram.

The searches yielded 1511 papers of which 21 RCTs were selected for full-text review. Three studies were excluded for not presenting a control, sham, or standard care group [28,29,30], two studies used multi-interventions from which the effects of CST could not be extrapolated [31,32], and one did not measure outcome variables evaluating the clinical effectiveness of CST [33]. Fifteen RCTs were thus included in the qualitative synthesis and seven were submitted for the quantitative synthesis. The description of the selection process is shown in the PRISMA flowchart diagram (Figure 1). 

Regarding the methodological quality of the studies evaluated with the PEDro scale, three studies were classified as low quality [34,35,36], eight studies as fair quality [37,38,39,40,41,42,43,44], and four as high quality [19,33,45,46] (Table 1). 

**Figure 2:**
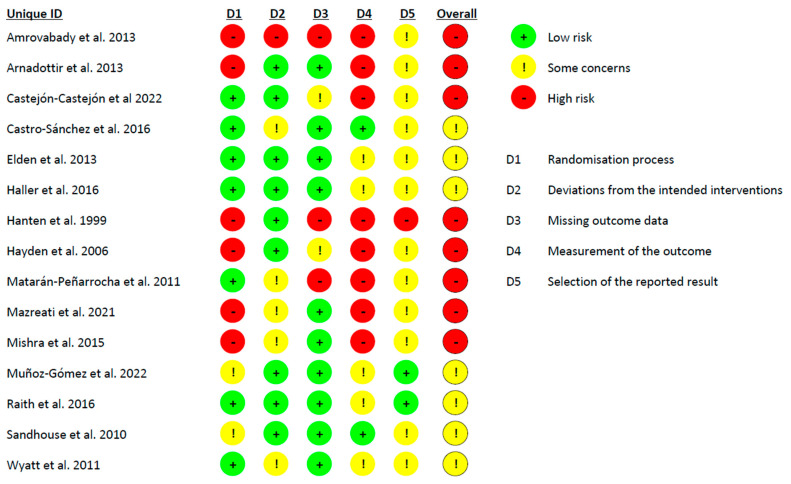
Cochrane Risk of Bias 2 tool [33,34,35,36,37,38,39,40,41,42,43,44,45,46,47].

The overall risk of bias was considered to be high for eight studies [34,35,36,37,39,40,41,43]. In the risk of bias tool, eight studies showed an unclear randomization process [34,36,37,38,39,41,43,46], and almost all the studies presented concerns about the measurement of the outcome variables [34,35,36,37,38,39,40,41,42,43,44,45,47] and about the selection of the reported results [34,35,36,37,39,40,41,43,44,45,46,47,48]. Figure 2 shows in detail the Cochrane Risk of Bias 2 tool results.

### 3.1. Clinical Effectiveness on Musculoskeletal Conditions

Eight RCTs were included, evaluating the clinical effectiveness of CST in patients with musculoskeletal conditions such as headache disorders, neck pain, low back pain, pelvic girdle pain, and fibromyalgia. Seven of them assessed pain intensity, and six assessed disability or impact. 

In the qualitative synthesis, six out of the seven studies assessing pain intensity reported statistically significant improvements in favor of the CST group [34,35,38,39,47,48]. Comparing the qualitative results to the minimum clinically important changes (MCID) described for each condition, none of the changes achieved were superior to the MCID described for headache disorders (2.5) [49], neck pain (2.1) [50], low back pain (1.5) [51], pelvic girdle pain (1.3) [52], or fibromyalgia (2.3) [53] (Table 1). Three out of the six studies assessing disability or impact reported statistically significant improvements in favor of the CST group [34,38,47]. Comparing the qualitative results to the MCIDs, the change achieved in headache impact was not superior to the MCID stated (5.5–8) [54,55]. Only Haller et al. [47] reported a change in the Neck Disability Index higher than the MCID (7) [56] (Table 1). 

In the quantitative synthesis, the certainty of evidence was very low on pain intensity (Appendix B). The meta-analysis provided a statistically significant but clinically insignificant difference in pain intensity in patients with headache disorders (mean difference (MD) −0.79 95% CI: −1.39 to −0.20, *I*^2^ 92%), and no benefits to low back pain (standardized mean difference (SMD) −1.68 95% CI: −3.89 to 0.52, *I*^2^ 93%) (Supplementary Figure). The certainty of the evidence was very low on headache impact (Appendix B), with no statistically significant effects for CST (SMD 0.02 95% CI: −0.44 to 0.48, *I*^2^ 93%) (Supplementary Figure).

### 3.2. Clinical Effectiveness for Non-Musculoskeletal Conditions

Seven studies evaluated the clinical effectiveness of CST in children with infantile colic, autism, attention deficit hyperactivity disorder, cerebral palsy, preterm infants, and patients with visual function deficits. 

In the qualitative synthesis, no statistically significant improvements were reported in patients with cerebral palsy, preterm infants, or patients with visual function deficits [42,44,46]. Four out of the seven studies reported statistically significant improvements in favor of the CST groups in children with infantile colic [40,41], autism [43], and deficit hyperactivity disorder [36] (Table 1). No MCIDs were found for the outcome variables assessed.

In the quantitative synthesis, the certainty of the evidence was very low in terms of crying and sleeping time of children with infantile colic (Appendix B). The meta-analysis showed no statistically significant results for crying time (MD −1.78 95% CI: −4.01 to 0.44, *I*^2^ 98%) and sleeping time (MD 1.77 95% CI: −0.12 to 3.66, *I*^2^ 90%) in infantile colic (Supplementary Figure).

### 3.3. High- Versus Low-Quality Studies

In general, the studies that had a lower risk of bias and higher scores on the PEDro scale showed no statistically significant differences between CST and control interventions. In contrast, the studies with higher risk of bias and lower PEDro scores suggested statistically significant differences in favor of CST. In musculoskeletal and non-musculoskeletal conditions, all the studies that had a PEDro score ≤ 6 and a high risk of bias showed statistically significant benefits in favor of CST. Studies with PEDro scores ≥ 6 and low risk of bias showed no benefits favoring CST. 

Only the outcome measure of pain intensity in RCTs of musculoskeletal conditions showed different results; those with a PEDro score ≤ 7 and high risk of bias generated positive but clinically insignificant changes in pain intensity in the CST groups. The only study that was an exception, scoring 8 points in the PEDro score and yielding a positive result, was the one by Haller et al. 

### 3.4. Adverse Events

Ten RCTs failed to mention adverse events. Five RCTs assessed adverse events, and all of them reported no serious adverse events [35,38,40,44,45]. 

## 4. Discussion

Our systematic review and meta-analysis were aimed at determining whether CST is clinically effective for musculoskeletal or non-musculoskeletal disorders. Fifteen RCTs were included in the qualitative and seven in the meta-analyses. For musculoskeletal disorders, the qualitative and quantitative synthesis suggested that CST produces no statistically significant or clinically relevant changes in pain and/or disability/impact in patients with headache disorders, neck pain, low back pain, pelvic girdle pain, or fibromyalgia. For non-musculoskeletal disorders, the qualitative and quantitative synthesis showed that CST was not effective in managing infant colic, preterm infants, cerebral palsy, or visual function deficits.

Several previous systematic reviews have investigated the effects of CST in different populations [1,15,18,19,57,58,59,60]. Most of them concluded that there was insufficient evidence to support CST in any condition. Our findings are thus in accordance with the previously published evidence [1,15,18,57,60]. Our systematic review and meta-analysis is the first that critically evaluates all the currently available evidence on CST in musculoskeletal and non-musculoskeletal conditions.

### 4.1. Musculoskeletal Conditions

In musculoskeletal conditions, despite the fact that most of the included studies showed statistically significant improvements in favor of the CST, the qualitative and quantitative syntheses showed that CST did not produce relevant clinical effects. Only Haller et al. reported clinically relevant changes in patients with neck pain. 

The validity of the results reported by some of the authors reporting positive results is, however, questionable. The studies that found statistically significant benefits in CST were not prospectively registered in any database [33,34,48], did not perform a concealed allocation [33,34,38], and did not use an intent-to-treat analysis, despite the fact that some of them presented a dropout rate higher than 15% [35,38,47,48]. Moreover, most of the studies were designed as single- or double-blind clinical trials, but all of them used a sham intervention without assessing the effectiveness of blinding. Finally, several studies assessed self-reported subjective outcome variables, which are open to reporting biases [33,34,38,48].

Haller et al. [47] reported clinically relevant changes and a PEDro score of 8; these findings should be interpreted with caution because of the limitations of this study: the study protocol was not prospectively registered. The authors described the method of patient blinding, but the success of the procedure was not evaluated. Furthermore, most of the outcome variables were self-reported, which carries a high risk of bias. In addition, there is a lack of clarity regarding patient assessments, the intervention, and the possibility of verbal and non-verbal interactions between the therapists that might impact the observed outcomes. 

### 4.2. Non-Musculoskeletal Conditions

In non-musculoskeletal conditions, CST was not effective for children with cerebral palsy and patients with visual function deficits. Two RCTs found statistically significant differences in favor of CST for infantile colic. However, in both studies, the parents were unblinded and were asked to fill in the diaries regarding crying and sleeping times. In addition, approximately 14% of the infants assigned to the control group were lost to follow-up, yet no intent-to-treat analysis was conducted [40,41]. Furthermore, the results of the quantitative synthesis showed no significant benefits, which is in accordance with previous systematic reviews and meta-analyses [60,61].

Two studies found statistically significant benefits of CST for children with autism and hyperactivity disorder [36,43]. However, no MCIDs were found to compare the results, and meta-analyses could not be performed because only one study was included for each condition. Neither study had prospectively registered the study protocol, randomized the participants correctly, and blinded the patients or the examiners. In addition, both studies used a small sample size. In the study by Mishra et al. [43], the parents received explanations about the benefits of CST; they then filled in the questionnaires, and no details were provided as to how the data were statistically analyzed. Therefore, these studies scored the lowest values in PEDro scores for non-musculoskeletal conditions. 

Generally speaking, the RCTs of non-musculoskeletal conditions had multiple methodological flaws. All the studies that found positive effects of CST were conducted in children. Parents want to help their children and tend to opt for CST after other interventions fail [3]. In these studies, they were asked to record the outcome variables without being blinded, which inevitably introduces bias. The RCTs by Wyatt et al. [44] and Raith et al. [42] were the only studies that described assessor blinding, and these trials both found no statistically significant effects of CST. 

Ten RCTs failed to mention adverse effects. Arguably, not reporting adverse effects in clinical trials constitutes a violation of research ethics [62]. The fact that the majority of trials completely neglected adverse effects can be seen as a reflection of the overall poor standards of research in this area. 

### 4.3. Implications for Clinical Practice

From a clinical perspective, CST is an intervention widely used by osteopaths, chiropractors, and some physiotherapists. It is included in the benchmarks for training in osteopathy. Yet in our evaluation of its clinical effectiveness, no good evidence supports its use in any condition. Our findings are in accordance with several previous systematic reviews [1,15,18]. In our view, this suggests that CST is not an evidence-based therapy. Therefore, it should not be used in clinical routine unless new robust evidence supporting its usefulness emerges. 

### 4.4. Limitations and Future Considerations

This systematic review and meta-analysis have several limitations. First, even though our literature searches were thorough, we can never be absolutely sure that no relevant studies have been missed. Second, the inclusion of many diverse conditions in one review complicates the interpretation of the results and might weaken the strength of our conclusions. Third, considerable heterogeneity exists across the included RCTs in terms of treatment duration and outcome variables. These factors might limit the validity of our quantitative syntheses. 

## 5. Conclusions

Our evaluation fails to show CST to be clinically effective for musculoskeletal or non-musculoskeletal disorders. Two RCTs suggested statistically significant benefits of CST in children. However, both studies are seriously flawed, and their findings are thus likely to be false positive. To date, no sound evidence supports the use of CST for any condition. Considering the biological implausibility of the concepts of CST, we feel that future studies in this area may not be warranted. If further research is nonetheless initiated, it should be conducted with improved methodological quality by registering the protocol prospectively, performing an adequate random allocation, ensuring participants and examiners are blinded, and including objective outcome measures. 

## Figures and Tables

**Table 1:** Qualitative synthesis of the results.

	Participants		Intervention					Outcome (Tool)	Main Results	PEDro Score
Author (Year)	Mean Age (SD)	Diagnosis	CST Group	Control Group	Session Duration	Frequency (Sessions/Week)	Total Number of Sessions			
Musculoskeletal disorders										
Headache disorders										
Hanten et al., 1999 A [34]	36 (12)	TTH	CST (*n* = 20)	Resting position (*n* = 20)	10 m	1 s/w	1	− Pain (VAS) − Impact (VAS)	ND ND	4
Hanten et al., 1999 B [34]	36 (12)	TTH	CST (*n* = 20)	Control (*n* = 20)	10 m	1 s/w	1	− Pain (VAS) − Impact (VAS)	↑ Pain ↑ Impact	4
Arnadottir et al., 2013 [37]	37.6 (9.3)	Migraine	CST (*n* = 10)	Control (*n* = 10)	NR	1.5 s/w	6	− Impact (HIT-6)	ND	5
Muñoz-Gómez et al., 2022 [38]	CST: 40.92 (7.95) CG: 37.64 (9.42)	Migraine	CST (*n* = 25)	Sham intervention (*n* = 25)	45 m	1 s/w	8	− Pain (VAS) − Migraine severity (HDI)	↑ Pain ↑ Severity	6
Neck pain										
Haller et al., 2016 [47]	CST: 44.2 (9.7) CG: 45.0 (10.5)	CNP	CST (*n* = 27)	Sham intervention (*n* = 27)	45 m	1 s/w	8	− Pain (VAS) − Neck disability (NDI)	↑ Pain ↑ Disability	8
Low back pain										
Castro-Sánchez et al., 2011 [33]	CST: 50 (11) CG: 53 (9)	CLBP	CST (*n* = 32)	Control (*n* = 32)	50 m	1 s/w	10	− Pain (VAS) − Disability (RMQ and ODI)	↑ Pain ND	7
Mazreati et al., 2021 [39]	CST: 34.28 (3.28) CG: 33.11 (3.20)	CLBP	CST (*n* = 30)	Control (*n* = 29)	30–45 m	NR	8	− Pain (McGill questionnaire)	↑ Pain	6
Pelvic girdle pain										
Elden et al., 2013 [45]	CST: 30.6 (3.9) CG: 31.3 (4.3)	Pregnant women with pelvic girdle pain	CST + standard care (*n* = 55)	Standard care (*n* = 57)	45 m	1 s/w	3	− Morning pain (VAS) − Evening pain (VAS) − Discomfort of pain (VAS) − Disability (DRI)	↑ Morning pain ND ND ND	8
Fibromyalgia										
Matarán-Peñarrocha et al., 2011 [35]	CST: 48.25 (13.34) CG: 52.26 (10.98)	Fibromyalgia	CST (*n* = 43)	Sham intervention (*n* = 41)	60 m	2 s/w	50	− Pain (VAS)	↑ Pain	4
Non-musculoskeletal conditions										
Infantile colic										
Castejón-Castejón et al., 2022 [40]	CST: 39.14 (20.15) days CG: 33.69 (15.14) days	Infantile colic	CST (*n* = 29)	Control (*n* = 25)	30–40 m	1 s/w	1 to 3	− Crying diary − Sleeping diary	↑ Crying ↑ Sleeping	6
Hayden et al., 2006 [41]	CST:46.4 (5.4) days CG: 44.5 (5.0) days	Infantile colic	CST (*n* = 14)	Control (*n* = 14)	30 m	1 s/w	4	− Crying diary − Sleeping diary	↑ Crying ↑ Sleeping	5
Preterm infants										
Raith et al., 2016 [42]	CST: 28 (25–33) weeks CG: 30 (27–33) weeks	Preterm infants	CST (*n* = 12)	Control (*n* = 13)	NR	2 s/w	6	− Motor Function (GMA, GMOS)	ND	5
Autism										
Mishra and Senapati 2015 [43]	CST: 3–10 CG: 3–10	Children with autism	CST + standard care (*n* = 10)	Standard care (*n* = 10)	60 m	5 s/w	40	− Autism evaluation (ATEC)	↑ Autism evaluation	5
Hyperactivity disorder										
Amrovabady et al., 2013 [36]	CST: 9.5 CG: 9.9	Attention deficit hyperactivity disorder	CST + standard care (*n* = 12)	Standard care (*n* = 12)	30 m	2 s/w	15	− Symptoms (CSI-4) − Behaviour (CPRS)	↑ Symptoms ↑ Behaviour	3
Cerebral palsy										
Wyatt et al., 2011 [44]	CST: 8.0 (5–12) CG: 7.6 (5–12)	Cerebral palsy	CST (*n* = 62)	Control (*n* = 67)	NR	1 s/month	6	− Motor function (GMFM66) − Physical function (CHQ) − Pain (PPP)	ND ND ND	6
Visual function										
Sandhouse et al., 2010 [46]	24.38 (3.03)	Patients with myopia, hyperopia, or astigmatism	CST (*n* = 15)	Sham intervention (*n* = 14)	5 m	1 s/w	1	− Distance visual acuity testing − Accomodative system testing − Local stereoacuity testing − Pupillary size testing − Retinoscopy − Vergence system testing	ND ND ND ↑ right pupillary size ND ND	7

↑ Statistically significant improvement. CST: craniosacral; CG: control group; TTH: tension-type headache; CNP: chronic neck pain; CLBP: chronic low back pain; NR: no reported; VAS: Visual Analog Scale; HIT-6: Headache Impact Test; HDI: Headache Disability Index; NDI: Neck Disability Index; RMQ: Roland Morris Questionnaire; ODI: Oswestry Disability Index; DRI: Disability Rating Index; GMA: General Movement Assessment; GMOS: General Movement Optimality Score; ATEC: Autism Treatment Evaluation Checklist; CSI-4: Children Severity Index; CPRS: Conners’ Parent Rating Scale; GMFM-66: Gross Motor Function Measure; CHQ: Child Health Questionnaire; PPP: Pediatric Pain Profile; ND: no difference.

## Data Availability

Not applicable.

## Supplementary Materials

The following supporting information can be downloaded at: https://www.mdpi.com/article/10.3390/healthcare12060679/s1, Supplementary Figure: Forest plot of the outcome variables.

## Appendix A. Detailed Search Strategy According to the PRISMA Model

PUBMED
Search strategy: (“osteopathic manipulation” [MeSH] OR “cranial mobilization” OR “craniosacral mobilization” OR “craniosacral manipulation” OR “cranial therapy” OR “craniosacral” OR “osteopathic cranial manipulative medicine” OR “cranial osteopathy” OR “craniosacral osteopathy” OR “cranial manipulation” OR “cranial field” OR “cranial osteopathic manipulative medicine” OR “osteopathic cranial manipulative medicine” OR “cranial manipulative medicine” OR “primary respiratory mechanism” OR “cranial rhythmic impulse” OR “fourth ventricular”)
Filter: clinical trial/randomized controlled trial
Data: 17 August 2023
Studies retrieved: 279
PEDRO
Search strategy: craniosacral
Data: 17 August 2023
Studies retrieved: 21
Search strategy: cranial osteopathy
Data: 17 August 2023
Studies retrieved: 7
Cochrane Library
Search strategy: (“osteopathic manipulation” OR “cranial mobilization” OR “craniosacral mobilization” OR “craniosacral manipulation” OR “cranial therapy” OR “craniosacral OR “osteopathic cranial manipulative medicine” OR “cranial osteopathy” OR “craniosacral osteopathy” OR “cranial manipulation” OR “cranial field” OR “cranial osteopathic manipulative medicine” OR “osteopathic cranial manipulative medicine” OR “cranial manipulative medicine” OR “primary respiratory mechanism” OR “cranial rhythmic impulse” OR “fourth ventricular”)
Data: 17 August 2023
Studies retrieved: 133
WOS
Search strategy: “osteopathic manipulation “ OR “cranial mobilization” OR “craniosacral mobilization” OR “craniosacral manipulation” OR “cranial therapy” OR “craniosacral” OR “osteopathic cranial manipulative medicine” OR “cranial osteopathy” OR “craniosacral osteopathy” OR “cranial manipulation” OR “cranial field” OR “cranial osteopathic manipulative medicine” OR “osteopathic cranial manipulative medicine” OR “cranial manipulative medicine” OR “primary respiratory mechanism” OR “cranial rhythmic impulse” OR “fourth ventricular”
Data: 17 August 2023
Studies retrieved: 988
OSTMED
Search strategy: “craniosacral therapy” OR “cranial osteopathy” OR “osteopathy in the cranial field” OR “osteopathic cranial manipulative medicine”
Data: 17 August 2023
Studies retrieved: 30

## Appendix B. Synthesis of Quantitative Results and Certainty of Evidence

**Outcome**	**No. of Studies (Participants)**	**Risk of Bias**	**Inconsistency**	**Imprecision**	**Indirectness**	**Publication Bias**	**Pooled Effect Estimate**	**Certainty of Evidence**
Headache disorders								
Pain intensity	2 (110)	Very serious ^a^	None	Serious ^c^	None	Begg test: 0.29 Egger test: 0.01	MD: −0.79 (−1.39, −0.20)	Very low
Headache impact	2 (60)	Very serious ^a^	None	Serious ^c^	Serious ^d^	No suspected	SMD: 0.02 (−0.44, 0.48)	Very low
Low back pain								
Pain intensity	2 (123)	Very serious ^a^	Very serious ^b^	Serious ^c^	None	No suspected	SMD: −1.68 (−3.89, 0.52)	Very low
Infant colic								
Crying time	2 (82)	Very serious ^a^	Very serious ^b^	Serious ^c^	Serious ^d^	No suspected	MD: −1.78 (−4.01, 0.44)	Very low
Sleeping time	2 (82)	Very serious ^a^	Very serious ^b^	Serious ^c^	Serious ^d^	No suspected	MD: 1.77 (−0.12, 3.66)	Very low
MD: mean difference; SMD: standardized mean difference. ^a^ Risk of bias was downgraded because more than 50% of the studies included presented fair or low methodological quality. ^b^ Inconsistency was downgraded because I^2^ was higher than 75%. ^c^ Imprecision was downgraded because the interventions were heterogeneous. ^d^ Indirectness was downgraded because the number of patients was <100.								
