# Supplementary material for: Is Craniosacral Therapy Effective? A Systematic Review and Meta-Analysis
Source: Healthcare (Basel). 2024 Mar 18;12(6):679. doi: 10.3390/healthcare12060679 (PMC10970181; doi:10.3390/healthcare12060679)
Supplement: Supplementary file 1 [file healthcare-12-00679-s001.zip › healthcare-2883502-supplementary.pdf]

Supplementary material: Forest plot of the outcome variables

Study or Subgroup	CST Mean	CST SD	CST Total	Sham or control Mean	Sham or control SD	Sham or control Total	Weight	Mean Difference IV, Random, 95% CI
Hanten et al.	2.16	1.81	10	2.67	2.33	20	15.4%	-0.51 [-2.03, 1.01]
Hanten et al. B	2.16	1.81	10	2.61	1.73	20	19.4%	-0.45 [-1.80, 0.90]
Muñoz-Gómez et al.	6.46	1.04	25	7.42	1.57	25	65.2%	-0.96 [-1.70, -0.22]
**Total (95% CI)**			**45**			**65**	**100.0%**	**-0.79 [-1.39, -0.20]**

Heterogeneity: Tau^2 = 0.00; Chi^2 = 0.58, df = 2 (P = 0.75); I^2 = 0%
Test for overall effect: Z = 2.60 (P = 0.009)

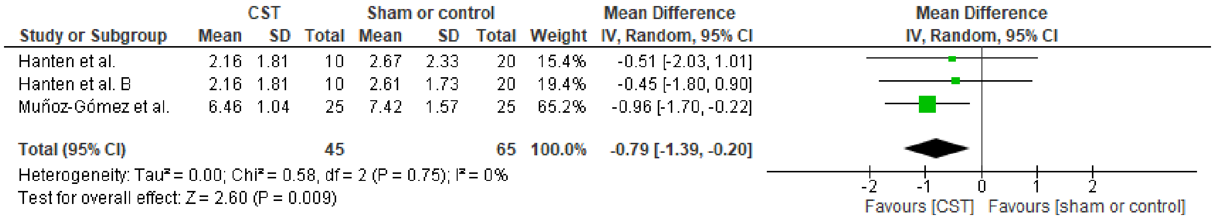

Figure S1. Forest plot of pain intensity in headache disorders [34,38].

Study or Subgroup	Std. Mean Difference	SE	Weight	Std. Mean Difference IV, Random, 95% CI
**3.1.2 Impact**				
Arnadottir et al.	-0.1649	0.4482	27.3%	-0.16 [-1.04, 0.71]
Hanten et al.	0.2383	0.3887	36.2%	0.24 [-0.52, 1.00]
Hanten et al. B	-0.0671	0.3874	36.5%	-0.07 [-0.83, 0.69]
**Subtotal (95% CI)**			**100.0%**	**0.02 [-0.44, 0.48]**
**Total (95% CI)**			**100.0%**	**0.02 [-0.44, 0.48]**

Subtotal: Heterogeneity: Tau^2 = 0.00; Chi^2 = 0.54, df = 2 (P = 0.76); I^2 = 0%
Test for overall effect: Z = 0.07 (P = 0.94)

Total: Heterogeneity: Tau^2 = 0.00; Chi^2 = 0.54, df = 2 (P = 0.76); I^2 = 0%
Test for overall effect: Z = 0.07 (P = 0.94)
Test for subgroup differences: Not applicable

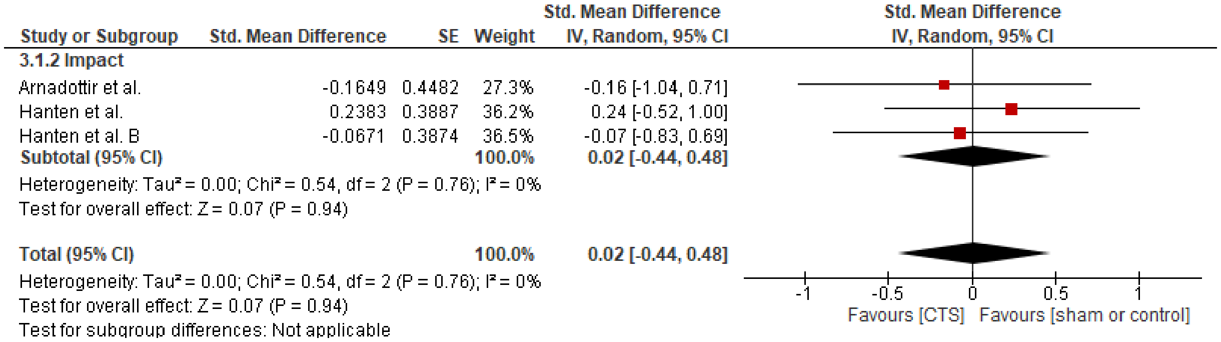

Figure S2. Forest plot of impact in headache disorders [34,37].

Study or Subgroup	Std. Mean Difference	SE	Weight	Std. Mean Difference IV, Random, 95% CI
Castro-Sanchez et al.	-0.5397	0.4572	49.3%	-0.54 [-1.44, 0.36]
Mazreati et al.	-2.7898	0.3689	50.7%	-2.79 [-3.51, -2.07]
**Total (95% CI)**			**100.0%**	**-1.68 [-3.89, 0.52]**

Heterogeneity: Tau^2 = 2.36; Chi^2 = 14.67, df = 1 (P = 0.0001); I^2 = 93%
Test for overall effect: Z = 1.49 (P = 0.14)

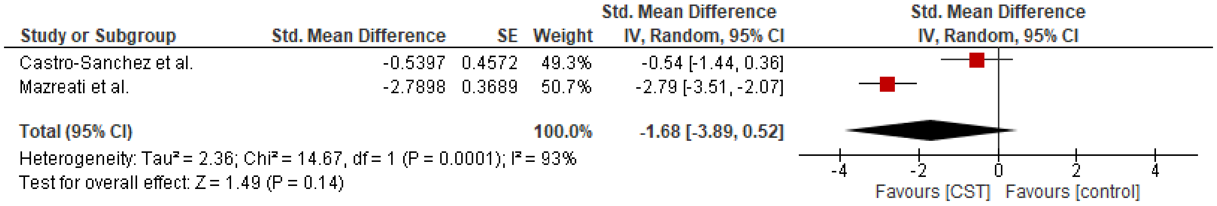

Figure S3. Forest plot of pain intensity in low back pain [33,39].

Study or Subgroup	Experimental Mean	Experimental SD	Experimental Total	Control Mean	Control SD	Control Total	Weight	Mean Difference IV, Random, 95% CI
**1.1.1 Craneosacral Therapy**								
Castejon-Castejon et al	0.017	0.09	29	2.96	1.7	25	49.0%	-2.94 [-3.61, -2.28]
Hayden et al	0.89	0.28	14	1.56	0.27	14	51.0%	-0.67 [-0.87, -0.47]
**Subtotal (95% CI)**			**43**			**39**	**100.0%**	**-1.78 [-4.01, 0.44]**
**Total (95% CI)**			**43**			**39**	**100.0%**	**-1.78 [-4.01, 0.44]**

Subtotal: Heterogeneity: Tau^2 = 2.52; Chi^2 = 40.78, df = 1 (P < 0.00001); I^2 = 98%
Test for overall effect: Z = 1.57 (P = 0.12)

Total: Heterogeneity: Tau^2 = 2.52; Chi^2 = 40.78, df = 1 (P < 0.00001); I^2 = 98%
Test for overall effect: Z = 1.57 (P = 0.12)
Test for subgroup differences: Not applicable

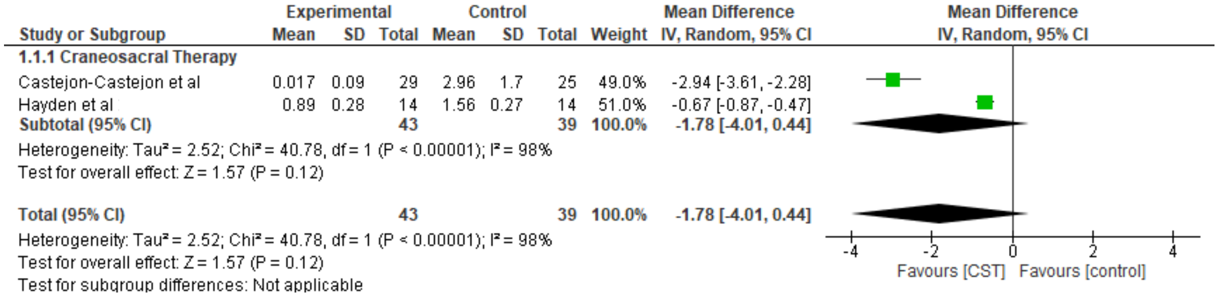

Figure S4. Forest plot of crying time in infantile colic [40,41].

Study or Subgroup	Experimental Mean	Experimental SD	Experimental Total	Control Mean	Control SD	Control Total	Weight	Mean Difference IV, Random, 95% CI
**1.1.1 Craneosacral Therapy**								
Castejon-Castejon et al	14.13	1.74	29	11.34	2.13	25	47.1%	2.79 [1.74, 3.84]
Hayden et al	12.9	0.58	14	12.04	0.79	14	52.9%	0.86 [0.35, 1.37]
**Subtotal (95% CI)**			**43**			**39**	**100.0%**	**1.77 [-0.12, 3.66]**
**Total (95% CI)**			**43**			**39**	**100.0%**	**1.77 [-0.12, 3.66]**

Subtotal: Heterogeneity: Tau^2 = 1.69; Chi^2 = 10.51, df = 1 (P = 0.001); I^2 = 90%
Test for overall effect: Z = 1.84 (P = 0.07)

Total: Heterogeneity: Tau^2 = 1.69; Chi^2 = 10.51, df = 1 (P = 0.001); I^2 = 90%
Test for overall effect: Z = 1.84 (P = 0.07)
Test for subgroup differences: Not applicable

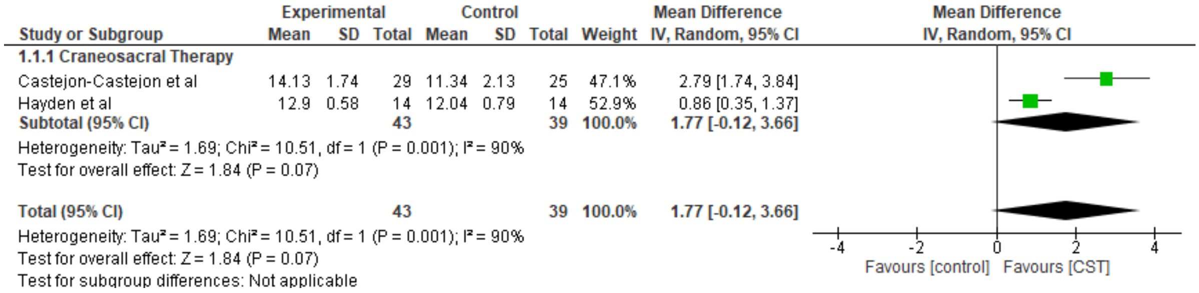

Figure S5. Forest plot of sleeping time in infantile colic [40,41].
